# A Longitudinal Change Patterns of Depression and Its Relationship with Socioeconomic Deprivation among Middle-Aged Adults in South Korea

**DOI:** 10.3390/ijerph182412957

**Published:** 2021-12-08

**Authors:** Soo-Bi Lee, Min-Ji Yu, Myeong-Sook Yoon

**Affiliations:** 1 Department of Social Welfare (BK21 FOUR), Jeonbuk National University, Jeonju 54896, Korea; sb88@jbnu.ac.kr (S.-B.L.); ymj@jbnu.ac.kr (M.-J.Y.); 2 Department of Social Welfare, Jeonbuk National University, Jeonju 54896, Korea

**Keywords:** midlife depression, socioeconomic deprivation, longitudinal change patterns, a latent class growth analysis, middle-aged Koreans

## Abstract

This study aimed to identify the longitudinal pattern changes of South Koreans’ midlife depression and determine the impact of socioeconomic deprivation on the observed change in patterns. In total, 3975 middle-aged individuals were examined by conducting a latent class growth analysis and multinomial logistic regression analysis on seven years of Korea Welfare Panel data (2012–2018) using STATA 16.0 (StataCorp LLC, College Station, TX, USA). The change patterns of midlife depression were classified into normal depression reduction group, mild depression maintenance group, and serious depression increase group. The impact of the experience of socioeconomic deprivation on the classified change patterns was examined using the normal depression reduction group as the reference group. It was found that the higher an individual’s nutritional, housing, occupational/economic, and healthcare deprivation, the higher their risk of mild depression maintenance or serious depression increase. The serious depression increase group showed higher relative risk ratios in all domains. Comprehensive and integrated social welfare services, such as stable income, housing welfare, and healthcare services, should be provided along with appropriate clinical interventions for depression alleviation that account for the pattern changes in midlife depression.

## 1. Introduction

In Korea, the middle-aged population (40–64 years) accounts for 40% of the total population [1]. In Korea’s aging society, middle-aged individuals are an important demographic in terms of providing social welfare. Consequently, social interest in middle adulthood has increased, particularly regarding the socioeconomic difficulties and mental health problems that they experience. Given the lowering of the retirement age due to the labor market structure, middle-aged Koreans’ work status has become unstable. They become vulnerable to multi-level stress due to the pressure of caring for their parents and dependent adult children, their growing financial and psychological burdens, and their swelling anxiety about their future as senior citizens [2,3,4,5]. The suicide rate among middle-aged Koreans is significantly higher in comparison to the OECD average [6]. According to the Health Insurance Review and Assessment Service, depressed individuals in their 40s and 50s account for 27.8% of the total depressed population [7]. Given the strong association between suicide and depression, which is a representative indicator of mental health [8,9,10], midlife depression is an important mental health problem that should not be overlooked.

Depression is a common emotional state that anyone can experience. Still, it is a severe problem among middle-aged Koreans because they tend to dismiss it as an insignificant negative mood until it develops into a chronic condition [11,12,13]. Moreover, the functional deterioration that middle-aged individuals undergo due to their physical and psychological changes, family relationships, and economic difficulties renders them prone to experiencing the emergence/escalation of depression compared to other age groups [14]. Changes in social role, social support, health status, life satisfaction, and posttraumatic stress disorder (PTSD) have been identified as major determinants of midlife depression [15,16,17,18]. Studies have also pointed out that poverty-related factors such as low socioeconomic status, low-income, and low education level are social causes of depression [19,20,21].

Depression-related socioeconomic factors such as socioeconomic status, income, education levels, and occupation are income-based poverty indicators. However, these indicators do not adequately reflect the social and cultural aspects associated with the various domains of socioeconomic deprivation that individuals are exposed to in everyday life [22,23,24,25,26,27]. Given that these indicators are income-oriented absolute measures that may diverge from individuals’ everyday experiences, it is necessary to consider a multidimensional approach to poverty and examine the deprivation experienced by individuals to clarify the relationship between poverty and midlife depression.

Recent studies have examined the association between depression and “deprivation”—a multidimensional measure of deficiencies that individuals experience in real life to break away from the traditional poverty-oriented approach. It involves an examination of individuals’ dietary life, living environment, working conditions, health status, education level, and social relationships [22,23,28,29,30,31,32,33]. It is important to examine the relationship between inequality and mental health because individual experiences of deprivation in multidimensional domains of life are diverse regardless of their income level. Moreover, deprivation can reflect individual experiences of deficiency [34,35,36,37]. Studies on deprivation and mental health often measure deprivation at the community level [30] and have inconsistently applied the concept of deprivation [23].

In addition, since the depression caused by socioeconomic difficulties varies based on one’s life cycle [14,38,39], existing studies are skewed toward older adults [40,41,42] and the adult population in general [23,43]. Thus, a limited number of studies have analyzed midlife depression. Furthermore, most of these studies followed a cross-sectional study design [27,33,44] or have been conducted based on latent growth modeling, which assumed the same developmental trajectories for all study participants [43,45]. This made it difficult to identify heterogeneous patterns of change in depression.

Therefore, this study intends to investigate the relationship between socioeconomic deprivation and the longitudinal change patterns of midlife depression while drawing on the findings of previous studies. Accordingly, this study sought to answer the following research questions: (1) What change patterns can midlife depression over time be classified into? (2) What effects do various domains of socioeconomic deprivation have on the observed longitudinal change patterns of midlife depression? This research results can be used to devise and implement appropriate intervention strategies to ultimately reduce the prevalence of depression among the middle-aged population.

## 2. Methods

### 2.1. Data and Sample

This study utilized seven years of data (2012–2018) from the Korea Welfare Panel. The Korea Welfare Panel—a nationwide panel survey—is an extremely useful resource for conducting a longitudinal study. It is an annual survey conducted by the Korean Institute for Health and Social Affairs, a state-run institution, in collaboration with the Social Welfare Institute of Seoul National University since 2006 (www.koweps.re.kr) (access on 25 November 2021) [46,47]. This survey has high representativeness and reliability because it includes small regional administrative units [46]. The analysis unit of this study was individuals, and the data from 3975 middle-aged adults (40–64 years) who were surveyed for seven consecutive years were analyzed. The low-income population group was intentionally oversampled for this study, and weighting was performed during the analysis to generalize the results.

### 2.2. Measures

#### 2.2.1. Outcome Variable: Depressive Symptoms

Depression was measured using the Center for Epidemiologic Studies Depression Scale (CES-D). The CES-D was developed to measure depression in the general public easily and has been used in many domestic and foreign studies [48]. Measurements on the CES-D scale have a standard format of 20 items, but have been developed into 11 questions in brief measurement forms [49,50]. The 11-version CES-D scale was proven almost identical to the reliability and validity of the 20-item standard scale [49]. The CES-D has good sensitivity, specificity, and high internal consistency for identifying the risk of depression [49,51]. In this 11-item depression scale, an individual’s psychological attitude and behavior domains are measured in a self-report format. Each item is rated on a four-point Likert scale (0–3), and the final score is calculated by summing the item scores [49]. Since a higher total score indicates a higher severity of depression, the total score itself was used as a continuous variable in this study.

#### 2.2.2. Predictive Factors: Socioeconomic Deprivation Factors

Socioeconomic deprivation was measured using the base year data from 2012. The society (state) to which individuals belong and its socioeconomic factors are reflected in deprivation [22,32,52]. Accordingly, this study reviewed the individual-oriented deprivation domains and measurement items that have been mainly used by Korean researchers [22,32,35]. Subsequently, the items for each of the following socioeconomic deprivation domains were finalized: nutritional deprivation (6 items), housing deprivation (10 items), educational deprivation (2 items), occupational/economic deprivation (4 items), social security deprivation (5 items), social deprivation (4 items), and healthcare deprivation (3 items). Each of these 34 items was designed as a binary variable (presence of experience = 1; absence of experience = 0). The number of deprivation experiences in each domain was summed and used for the analysis. The detailed items of each deprivation domain were as follows: the nutritional deprivation domain included items that queried the participants’ experience of skipping meals (oneself or family) or eating less than their appetite due to a lack of funds. The housing deprivation domain checked whether the participants’ residences meet basic and essential housing standards such as safety, basement or rooftop dwelling, enough rooms, and sufficient space for the household. The educational deprivation domain included items that queried the participants’ experience of suspending their education or their children’s education due to economic difficulties. The occupational/economic deprivation domain measured the participants’ total cost of living (whether it exceeded the minimum standard), unemployment status, and experiences of working in a hazardous environment. The social security deprivation domain measured the participants’ insurance subscription status (pension, industrial accident compensation, unemployment, and healthcare). The social deprivation domain measured their satisfaction with their social relationships with family and acquaintances and their experiences of being helped financially. The healthcare deprivation domain included items that queried their experiences of neglecting treatment due to their inability to pay for hospital treatment (Table S1).

#### 2.2.3. Demographic Characteristics

The sociodemographic variables in this study included gender (male = 0, female = 1), religion (affirmative = 0, negative = 1), and marital status. Married, bereaved, divorced, separated, and unmarried was re-coded into three categories, i.e., married/unmarried/bereaved, divorced, and separated. The five categories of regions, i.e., Seoul, metropolitan cities, cities, counties, and urban and rural hybrids were re-coded into two categories and used for analysis: location (capital area = 1, non-capital area = 0), income level (general = 1, low-income group = 0), and education level (≤middle school, high school, ≥college).

### 2.3. Statistical Analysis

We conducted a latent class growth analysis and multinomial logistic regression analysis using STATA 16.0 software to identify the longitudinal change patterns of midlife depression and test the impact of socioeconomic deprivation on these change patterns, respectively.

Unlike latent growth modeling, which analyzes a group’s change trajectory on a single frame, latent class growth analysis is useful for classifying different latent trajectory classes within a group [53]. The researcher does not assume any change patterns in advance but objectively classifies the heterogeneous latent classes based on the participants’ response values. The researcher also tests whether the pattern change shown by individuals included in the heterogeneous classes is statistically significant [54].

Then, the goodness-of-fit for the classified latent classes is tested to identify the model that has classified the optimal number of latent classes (change patterns). In this process, a model with K latent classes, which has the smallest value compared to Akaike’s information criterion (AIC) and Bayesian information criterion (BIC) with the model with K-1 latent classes, has the best model fit. On this note, any latent class containing less than 5% of the participants can be excluded [55]. However, because this type of index is sensitive to the sample size, the final number of classes should be determined by considering the research questions set for the study and simplicity of analysis, in addition to the fit index, classification quality, and model comparison [56,57]. Therefore, after verifying the values of AIC and BIC as model-data fit indices and the member ratio of each classified class, we determined the optimal number of change patterns of midlife depression by considering the probability and simplicity of analysis. Then, the impact of socioeconomic deprivation on the classified change patterns was verified using multinomial logistic regression analysis. At this time, the results of the polynomial logistic analysis were presented as a relative risk ratio. This is an odds ratio that can be compared between alternatives [58], and when the multinomial logistic analysis is conducted in the STATA program, this value is generated.

## 3. Results

### 3.1. Participants’ Demographic Characteristics

The participants’ demographic characteristics were analyzed based on values from the reference year (2012) (Table 1). Women slightly outnumbered men (53.0% vs. 27.0%) in the sample. In total, 41.8% of the participants graduated from high school, 30.2% had a college degree or higher, and 28.1% only attended middle school at most. Most of the participants were married (84.1%), whereas divorced/widowed/separated and unmarried participants accounted for 12.6% and 3.3% of the sample, respectively. A higher number of participants had a religious affiliation (55.0% vs. 45.0%), and a slightly higher number of participants lived in the capital area than non-capital areas (52.5% vs. 47.5%). The income-based poverty ratio of general households to low-income households was 86.2%:13.8%.

### 3.2. Intervariable Correlation

A correlation analysis was performed to verify the intervariable correlations. The correlation coefficients ranged between 0.002 and 0.359 and did not exceed 0.8 (Table S2).

### 3.3. Classification of the Change Patterns of Midlife Depression (Latent Classes of Developmental Trajectories)

A latent class growth analysis was performed to classify the change patterns of midlife depression. Four classified change patterns were modeled, as shown in Table 2. A comparison of the fit indices of the individual models revealed that the values of the BIC and AIC—the relative fit indices—decreased when the number of change patterns increased by one. The models that did not exceed the cut-off ratio of 5% of the participants were eliminated. The remaining three models were selected after verifying that the classification models for the three change patterns matched the analysis results.

**Figure 1:**
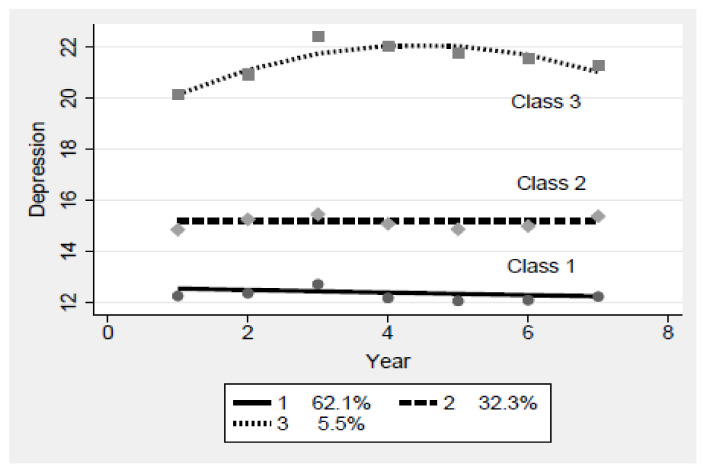
Change patterns of midlife depression.

The developmental trajectories and statistical significance of the three change patterns of the selected classification models were verified (Figure 1, Table 3). In addition, each type of change was named based on the characteristics of the change patterns. The first change pattern (Class 1) was the group with the lowest level of depression, which showed a linearly decreasing tendency over time. In total, 62.1% of the midlife depression cases belong to this group. In consideration of these characteristics, this group was named the “normal depression reduction group.” The group whose CES-D score remained at 16 points or less without change was the second change pattern (Class 2). In total, 32.3% of the midlife depression cases belong to this group. Thus, this group was named the “mild depression maintenance group.” The third change pattern (Class 3) showed a non-linear trend with a very high baseline value, which increased over time and slightly decreased in the last three waves. In total, 5.5% of the midlife depression cases belonged to this group. Since the decrease in the later phase was not significant, this group was named the “serious depression increase group.”

### 3.4. Verification of the Socioeconomic Deprivation Domains That Affect the Change Patterns of Midlife Depression

Three patterns of midlife depression were identified in Section 3.3. Considering their change patterns in comparison to the baseline values, we named them “normal depression reduction,” “mild depression maintenance,” and “serious depression increase.” To verify the impact of socioeconomic deprivation on these three change patterns, we set the mild depression maintenance group as the reference group, performed a multinomial logistic regression analysis, and presented their respective relative risk ratios (RRR) (Table 4). The model fit of the analysis was established with a statistically significant LR Chi^2^ value, and the explanatory power of the model was expressed with the Pseudo R^2^ value (0.166). Finally, the effects of socioeconomic deprivation in seven deprivation domains on the change patterns of midlife depression were verified.

Gender, marital status, and income level were the socioeconomic variables that affected the change patterns of midlife depression. Compared to women, men had a 1.509-fold (*RRR* = 1.509, *p* < 0.001) higher risk of belonging to the mild depression maintenance group and a 2.930-fold (*RRR* = 2.930, *p* < 0.001) higher risk of belonging to the serious depression increase group than to the mild depression maintenance group. Compared to the married group, the divorced/widowed/separated group had a higher risk of belonging to the mild depression maintenance and serious depression increase groups than the normal depression reduction group. Compared to the non-poverty group, the low-income group had a 1.868-fold (*RRR* = 1.868, *p* < 0.001) higher risk of belonging to the mild depression maintenance group and a 5.251-fold (*RRR* = 5.251, *p* < 0.001) higher risk of belonging to the serious depression increases group than the normal depression reduction group. However, education level, religion, and location showed statistically significant differences only in the mild depression maintenance group. A higher education level was associated with a lower risk of belonging to the mild depression maintenance group than those who had only attended middle school at most. The group with a religious affiliation had a 0.805-fold lower risk of belonging to the mild depression maintenance group than the group without a religious affiliation. Compared to the participants who lived in non-capital areas, those who lived in the capital area had a 1.284-fold (*RRR* = 1.284, *p* < 0.05) higher risk of belonging to the mild depression maintenance group than the normal depression reduction group.

Second, nutritional, housing, occupational/economic, and healthcare deprivation were the socioeconomic deprivation domains that affected the change patterns of midlife depression. The higher an individual’s nutritional deprivation, the higher their risk of belonging to the mild depression maintenance group [2.024-fold (*RRR* = 2.024, *p* < 0.01)] or serious depression increase group [3.232-fold (*RRR* = 3.232, *p* < 0.001)] than the normal depression reduction group. The higher an individual’s housing deprivation, the higher their risk of belonging to the mild depression maintenance group [1.178-fold (*RRR* = 1.178, *p* < 0.001)] or serious depression increase group [1.319-fold (*RRR* = 1.319, *p* < 0.001)]. The higher an individual’s occupational/economic deprivation, the higher their risk of belonging to the mild depression maintenance group [1.421-fold (*RRR* = 1.421, *p* < 0.001)] or serious depression increase group [1.669-fold (*RRR* = 1.669, *p* < 0.001)] than the normal depression reduction group. Finally, the higher an individual’s healthcare deprivation, the higher their risk of belonging to the mild depression maintenance group [2.310-fold (*RRR* = 2.310, *p* < 0.001)] or serious depression increase group [5.303-fold (*RRR* = 5.303, *p* < 0.001)] than the normal depression reduction group.

In particular, the RRR values of the nutritional, housing, occupations/economic, and healthcare deprivation domains, which adversely affected the change patterns of midlife depression, were greater in the serious depression increase group than in the mild depression maintenance group.

## 4. Discussion

In this study, we performed a latent class growth analysis and multinomial logistic regression analysis to verify the impact of socioeconomic deprivation on the longitudinal change patterns of the depression experienced by middle-aged Koreans. The following implications can be drawn from the results of this study.

First, three patterns of midlife depression were identified. In consideration of the characteristics of these change patterns, they were named “normal depression reduction,” “mild depression maintenance,” and “serious depression increase.” Unlike the results of previous longitudinal research that assumed the same change pattern in all participants [43], the results of this study revealed that midlife depression is manifested in various change patterns. Therefore, when devising intervention strategies for mitigating midlife depression, programs tailored to such change patterns need to be administered instead of applying a uniform approach based on public health. In particular, health promotion is not limited to the responsibility of the health sector alone. Health should be regarded as an important resource for social, economic, and personal development; it is necessary to establish an institutional delivery system that regards health as a concept of social investment as part of a healthy public policy [59]. For example, individuals belonging to the normal depression reduction group and the mild depression maintenance group, which are within the normal range of depression, could be provided with preventive education and intervention programs in the community mental health service system. Additionally, those in the mild depression maintenance group would need in-depth interventions such as stress coping or psychological/emotional support programs to address their long-term mild symptomology. On the other hand, those in the serious depression increase group require early interventions such as the spread of professional mental health counselors at work, where they spend most of their daily lives. It is paramount to establish a system to integrate medical treatment into mental health promotion programs designed to improve chronic depression and vulnerable mental health while accounting for the changing pattern of these conditions.

Second, the analysis of the socioeconomic deprivation domains that affect the change patterns of midlife depression revealed that the higher an individual’s nutritional deprivation, housing deprivation, occupational/economic deprivation, and healthcare deprivation, the higher their risk of belonging to the mild depression maintenance or serious depression increase groups than the normal depression reduction group. Additionally, the serious depression increase group presented higher RRR in all domains. This result can underpin the World Health Organization’s model of social health determinants [60] and the model of health determinants of Dahlgren and Whitehead [61], which are the theoretical basis for preparing policies to address health inequality. In other words, it was a reaffirmation that safe housing, social support networks, bonds, physical environment, employment environment, and occupational conditions affect people’s health and well-being. In particular, these results support those of previous studies that fragmentarily examined the correlations between depression and deprivation in terms of diet, housing environment, occupational/economic status, and healthcare [26,62]. However, the results regarding social deprivation (social relations and social support), which was reported as an important predictor of depression, were not consistent with the results of previous research [63]. Based on these research results, there is a need to prepare a public policy system and provide services to maintain a healthy life for the middle-aged at the society-wide level through the lens of health inequality, not just clinical intervention in depression and mental health. Specifically, it is necessary to establish a service delivery system to actively identify middle-aged adults who are experiencing severe nutritional, housing, occupational/economic, and healthcare deprivation and provide them with continuous monitoring and support to improve their mental health. Depression- and mental-health-related clinical interventions will also have to be linked with comprehensive and integrated social welfare services. These services should include income support, stable employment, housing welfare, and healthcare services, to ensure a better quality of life for such individuals.

The significance of this study is that it extended income-oriented poverty indicators such as schooling, educational attainment, and income level to various domains of deprivation that middle-aged adults experience in their daily lives. Additionally, it used their experiences of deprivation as the analysis variables. It also examined midlife depression by taking into account various longitudinal change patterns derived through a latent class growth analysis. Nevertheless, it has limitations in several aspects. First, deprivation indicators were constructed only within the questions available from the use of secondary data. Accordingly, all practical deprivation experiences that can occur in various life areas were not examined. Second, the pattern of change in socioeconomic deprivation experience was not considered. Therefore, follow-up studies should examine the influence that the changes in socioeconomic deprivation experience have on the types of change in depression. It is expected that a more dynamic process can be identified, and more diverse discussions can be made.

## 5. Conclusions

In this study, we examined the association between various types of socioeconomic deprivation experienced by middle-aged adults and the change patterns of their midlife depression. We derived three major change patterns of midlife depression: normal depression reduction, mild depression maintenance, and serious depression increase. Nutritional, housing, healthcare, and occupational/economic deprivation significantly affected these changes in patterns, and the relative risk ratio increased as the depression score moved toward the serious depression increase group. The practical implications of these results are twofold. First, it provides basic data for devising intervention strategies that account for the change patterns of midlife depression. Second, it is necessary to provide both a mental health policy tailored to the middle-aged and a socioeconomic support policy for mental health services. Namely, it explained the importance of providing social welfare services, such as stable income, employment, housing welfare, and healthcare, in tandem with clinical interventions to improve the depression symptomology and mental health of the middle-aged. Future research should analyze the changes in their experiences of socioeconomic deprivation.

## Figures and Tables

**Table 1:** The participants’ sociodemographic characteristics (*n* = 3975).

Variable	Attribute	Frequency (*n*)	Ratio (%) *
Gender	Male	1698	47.0
	Female	2277	53.0
Education level	≤Middle school	1536	28.1
	High school	1601	41.8
	≥College	838	30.2
Marital status	Married	3162	84.1
	Unmarried	170	3.3
	Div./Wid./Sep.	643	12.6
Religion (Y/N)	Yes	2204	55.0
	No	1771	45.0
Location	Capital area	1471	47.5
	Non-capital area	2504	52.5
Poverty	Non-poverty (general)	3112	86.2
	Low-income	863	13.8
Variable	Survey period	Mean	S.E.
Depression	Wave 1 (2012)	13.5	3.75
	Wave 2 (2013)	13.8	3.98
	Wave 3 (2014)	14.2	3.99
	Wave 4 (2015)	13.7	4.08
	Wave 5 (2016)	13.6	3.95
	Wave 6 (2017)	13.6	3.79
	Wave 7 (2018)	13.8	4.06

* Application of ratio weight.

**Table 2:** Classification of the latent classes (change types) of the developmental trajectories of depression.

Latent Class (*n*)	Goodness-of-Fit		Ratio to Belong to a Latent Class (%)			
	BIC	AIC	1st	2nd	3rd	4th
1	−62,869.5	−60,856.9	100.0			
2	−58,796.2	−58,777.3	81.93	18.07		
3	−58,346.5	−58,318.3	62.11	32.34	5.55	
4	−58,204.2	−58,166.5	42.48	45.51	10.74	1.27

**Table 3:** Estimates of the latent class models for midlife depression developmental trajectories.

Class	Parameter	Estimate	S.E.	Name of the Latent Class
1	Intercept	10.452 ***	0.222	Normal depression reduction group
	Linear	−0.123 **	0.036	
2	Intercept	14.594 ***	0.561	Mild depression maintenance group
3	Intercept	18.790 ***	0.999	Serious depression increase group
	Linear	1.472 **	0.505	
	Quadratic	−0.165 **	0.059	

** *p* < 0.01, *** *p* < 0.001.

**Table 4:** Impact of socioeconomic deprivation on the change patterns of midlife depression.

Variable	Normal Depression Reduction Group (Reference)			
	Mild Depression Group		Serious Depression Group	
	RRR	Robust SE	RRR	Robust SE
**Intercept**	0.150 ***	0.028	0.004 ***	0.001
**Gender (female)**	1.509 ***	0.153	2.930 ***	0.623
**Education level**				
High school	0.748 **	0.081	0.831	0.172
College or higher	0.688 **	0.095	1.015	0.285
**Marital status (married)**				
Unmarried	1.928 **	0.455	5.232 ***	1.888
Divorced/widowed/separated	1.790 ***	0.248	3.334 ***	0.730
**Religion (Yes)**	0.805 *	0.079	0.770	0.149
**Income (typical household)**	1.868 ***	0.236	5.251 ***	1.100
**Region (capital area)**	1.284 *	0.125	1.433	0.274
**Nutritional deprivation**	2.024 **	0.413	3.232 ***	0.718
**Housing deprivation**	1.178 ***	0.045	1.319 ***	0.074
**Educational deprivation**	1.223	0.344	1.050	0.487
**Social security deprivation**	1.069	0.045	0.942	0.081
**Economic deprivation**	1.421 ***	0.130	1.669 **	0.254
**Social deprivation**	1.135	0.108	0.884	0.142
**Healthcare deprivation**	2.310 ***	0.217	5.303 ***	1.119

*n* = 3975, LR chi^2^ (30) = 542.8, *p* = 0.000 Pseudo R^2^ = 0.166. * *p* < 0.05, ** *p* < 0.01, *** *p* < 0.001.

## Supplementary Materials

The following are available online at https://www.mdpi.com/article/10.3390/ijerph182412957/s1, Table S1: Items pertaining to each socioeconomic deprivation domain; Table S2: Main variable correlations.

## References

[1] Statistics Korea. https://www.kostat.go.kr/portal/korea/kor_nw/1/6/8/index.board?bmode=read&bSeq=&aSeq=386979&pageNo=1&rowNum=10&navCount=10&currPg=&searchInfo=&sTarget=title&sTxt=.

[2] Yoo T.K., Kang S.M., Jeong C. (2014). A study on factors affecting the likelihood of the poverty exit and entry of the asset-poor middle aged and elderly. Korean J. Soc. Welf. Stud..

[3] Lee Y. (2013). Preparation characterizations for old age of the baby boomers. J. Korea Content Assoc..

[4] Kang S.R., Moon S.H. (2011). A study on policy design for quality of life of mid-elderly and elderly population over age 45: Evidence from KLoSA. J. Korean Assoc. Policy Stud..

[5] Lee Y. (2017). Providing family support and depression: Focusing on babyboom women. J. Fam. Relat..

[6] Song S.Y. (2016). The relationship among the social exclusion, depression, and suicidal ideation in middle-aged individuals. Men’s Health Soc. Work.

[7] Health Insurance Review & Assessment Service (HIRA) Citizen-Centered Smart HIRA System. https://opendata_visual.hira.or.kr/hbis/dissAction.do?keywd=%EC%9A%B0%EC%9A%B8%EC%A6%9D&type=1.

[8] Kessler R.C., Chiu W.T., Demler O., Walters E.E. (2005). Prevalence, severity, and comorbidity of 12-Month DSM-IV disorders in the National Comorbidity Survey replication. Arch. Gen. Psychiatry.

[9] Hawton K., Comabella C.C., Haw C., Saunders K. (2013). Risk factors for suicide in individuals with depression: A systematic review. J. Affect. Disord..

[10] Lee S.B., Chung S., Lee H., Seo J.S. (2018). The Mutual Relationship between Men’s Drinking and Depression: A 4-Year Longitudinal Analysis. Alcohol Alcohol..

[11] Jeon H.J., Walker R.S., Inamori A., Hong J.P., Cho M.J., Baer L., Clain A., Fava M., Mischoulon D. (2014). Differences in depressive symptoms between Korean and American outpatients with major depressive disorder. Int. Clin. Psychopharmacol..

[12] Kwon H., Yoon K.L., Joormann J., Kwon J.H. (2013). Cultural and gender differences in emotion regulation: Relation to depression. Cogn. Emot..

[13] Lee H., An S. (2019). Mediating effects of emotional venting via Instant Messaging (IM) and positive emotion in the relationship between negative emotion and depression. J. Korean Acad. Community Health Nurs..

[14] Mirowsky J., Ross C.E. (1992). Age and depression. J. Health Soc. Behav..

[15] Tanaka H., Sasazawa Y., Suzuki S., Nakazawa M., Koyama H. (2011). Health status and lifestyle factors as predictors of depression in middle-aged and elderly Japanese adults: A seven-year follow-up of the Komo-Ise Cohort study. BMC Psychiatry.

[16] Stafford M., McMunn A., Zaninotto P., Nazroo J. (2011). Positive and Negative Exchanges in Social Relationships as Predictors of Depression: Evidence from the English Longitudinal Study of Aging. J. Aging Health.

[17] Brockmann H. (2010). Why are middle-aged people so depressed? Evidence from West Germany. Soc. Indic. Res..

[18] Koivumaa-Honkanen H., Kaprio J., Honkanen R.J., Viinamäki H., Koskenvuo M. (2004). Life satisfaction and depression in a 15-year follow-up of healthy adults. Soc. Psychiatry Psychiatr. Epidemiol..

[19] Hudson C.G. (2005). Socioeconomic Status and Mental Illness: Tests of the Social Causation and Selection Hypotheses. Am. J. Orthopsychiatry.

[20] Belle D. (1990). Poverty and women’s mental health. Am. Psychol..

[21] Phelan J.C., Link B.G., Tehranifar P. (2010). Social Conditions as Fundamental Causes of Health Inequalities: Theory, Evidence, and Policy Implications. J. Health Soc. Behav..

[22] Heo J.H., Cho Y.T., Kwon S.M. (2010). The effects of socioeconomic deprivations on health. Korean J. Sociol..

[23] Kim J.H., You J.W., Song I.H. (2015). Effects of socioeconomic deprivation on depressive mood: Analysis of the moderating effect of age. Health Soc. Welf. Rev..

[24] Townsend P. (1979). Poverty in the United Kingdom: A Survey of Household Resources and Standards of Living.

[25] Kim T., Lee J.M., Jung J.W. (2015). A study of elderly poverty and depression: Focusing on the multidimensional concept of poverty. Health Soc. Welf. Rev..

[26] Kuruvilla A., Jacob K.S. (2007). Poverty, social stress & mental health. Indian J. Med. Res..

[27] Salmond C., Crampton P., King P., Waldegrave C. (2006). NZiDep: A New Zealand index of socioeconomic deprivation for individuals. Soc. Sci. Med..

[28] Fernández-Niño J.A., Manrique-Espinoza B.S., Bojorquez-Chapela I., Salinas-Rodríguez A. (2014). income inequality, socioeconomic deprivation and depressive symptoms among older adults in Mexico. PLoS ONE.

[29] Gunnell D.J., Peters T.J., Kammerling R.M., Brooks J. (1995). Relation between parasuicide, suicide, psychiatric admissions, and socioeconomic deprivation. BMJ.

[30] Burrows S., Auger N., Gamache P., St-Laurent D., Hamel D. (2011). Influence of social and material individual and area deprivation on suicide mortality among 2.7 million Canadians: A prospective study. BMC Public Health.

[31] Law C., Snider A., Leo D.D. (2014). The influence of deprivation on suicide mortality in urban and rural Queensland: An ecological analysis. Soc. Psychiatry Psychiatr. Epidemiol..

[32] Lee W., Im R. (2014). Study for the relationship between perceptions of inequality and deprivation in Korea: Focusing on the mediating role of depression. Health Soc. Welf. Rev..

[33] Lee S.B., Chung S., Lee S. (2016). Economic deprivation and problem drinking: Mediating effect of perceived income inequality and depressive mood. Alcohol Health Behav. Res..

[34] Pérez-Mayo J. (2003). Measuring Deprivation in Spain, IRISS Working Paper Series 2003–09, IRISS at CEPS/INSTEAD. https://ideas.repec.org/p/irs/iriswp/2003-09.html.

[35] Yeo E. (2020). Impact of deprivation experience on social integration perceptions: Centered on differences by age group. Survey Res..

[36] Marmot M. (1997). Inequality, deprivation and alcohol use. Addiction.

[37] Wilkinson R.G., Pickett K.E. (2007). The problems of relative deprivation: Why some societies do better than others. Soc. Sci. Med..

[38] Levecque K., Van Rossem R., De Boyser K., Van de Velde S., Bracke P. (2011). economic hardship and depression across the life course: The impact of welfare state regimes. J. Health Soc. Behav..

[39] Alwin D.F., Wray L.A. (2005). A Life-Span Developmental Perspective on Social Status and Health. J. Gerontol. B Psychol. Sci. Soc. Sci..

[40] Kim B.K., Ha Y.J., Choi S. (2014). A vertical study on the factors which are influenced on depression of the aged: Focusing on physical, psychological, social factors. J. Korea Gerontol. Soc..

[41] Kim S.Y., Heo S.H., Chang S.J. (2018). The effects of socioeconomic deprivation on health status in the elderly: Focusing on the mediating role of depression. Health Soc. Welf. Rev..

[42] Cheung K.C.K., Chou K.L. (2019). Poverty, deprivation, and depressive symptoms among older adults in Hong Kong. Aging Ment. Health.

[43] Son Y.A. (2018). Study on the influence of socioeconomic deprivation on depression: Focusing on latent growth modeling analysis. JKDAS.

[44] Yeo E.G. (2020). The effect of material deprivation on depression: Focused on effect by life cycle and area of deprivation. Health Soc. Welf. Rev..

[45] Kahng S.K., Jung E.H., Kim B. (2015). Inequalities in the trajectory of depressive symptoms and its associated factors: Using the Korean Welfare Panel Study from 2006 to 2013. Korean J. Soc. Welf. Res..

[46] Seoul National University (SNU) (2019). Korean Welfare Panel Study User’s Guide.

[47] Yoo K.B., Park E.C., Jang S.Y., Kwon J.A., Kim S.J., Cho K.H., Kim J.H., Park S. (2016). Association between employment status change and depression in Korean adults. BMJ Open.

[48] Shin J., Lee T., Yun S.J. (2017). A bifactor approach to the factor structure study of the CES-D scale. Korean J. Stress Res..

[49] Kohout F.J., Berkman L.F., Evans D.A., Cornoni-Huntley J. (1993). Two shorter forms of the CES-D depression symptoms index. J. Aging Health.

[50] Radloff L.S. (1977). The CES-D scale: A self-report depression scale for research in the general population. Appl. Psychol. Meas..

[51] Lewinsohn P.M., Seeley J.R., Roberts R.E., Allen N.B. (1997). Center for epidemiologic studies depression scale (CES-D) as a screening instrument for depression among community-residing older adults. Psychol. Aging.

[52] Joung W.O. (2008). A study on the defining method about deprivation and participantive poverty. Proceedings of the Korean Welfare Panel Conference, Seoul, Korea, 26 September 2008.

[53] Andruff H., Carraro N., Thompson A., Gaudreau P., Louvet B. (2009). Latent Class Growth Modelling: A Tutorial. Tutor. Quant. Methods Psychol..

[54] Muthén B., Muthén L.K. (2000). Integrating person-centered and variable-centered analyses: Growth mixture modeling with latent trajectory classes. Alcohol. Clin. Exp. Res..

[55] Muthén B., Shedden K. (1999). Finite mixture modeling with mixture outcomes using the em Algorithm. Biometrics.

[56] Jung T., Wickrama K.J.S. (2008). An introduction to latent class growth analysis and growth mixture modeling. Soc. Personal. Psychol. Compass.

[57] Nylund K.L., Asparouhov T., Muthén B.O. (2007). Deciding on the number of classes in latent class analysis and growth mixture modeling: A monte carlo simulation study. Struct. Equ. Model..

[58] Cameron A.C., Trivedi P.K. (2009). Microeconometrics Using Stata.

[59] World Health Organization (WHO). http://www.who.int/healthpromotion/conferences/previous/ottawa/en/.

[60] Centers for Disease Control and Prevention (2010). Establishing a Holistic Framework to Reduce Inequities in HIV, Viral Hepatitis, STDs, and Tuberculosis in the United States.

[61] Dahlgren G., Whitehead M. (1991). Policies and Strategies to Promote Social Equity in Health.

[62] Lee S.H. (2020). An analysis of the relationship between housing poverty and problematic drinking using panel data. Health Soc. Welf. Rev..

[63] Grav S., Hellzèn O., Romild U., Stordal E. (2012). Association between social support and depression in the general population: The HUNT study, a cross-sectional survey. J. Clin. Nurs..

