# Supplementary material for: A Longitudinal Change Patterns of Depression and Its Relationship with Socioeconomic Deprivation among Middle-Aged Adults in South Korea
Source: Int J Environ Res Public Health. 2021 Dec 8;18(24):12957. doi: 10.3390/ijerph182412957 (PMC8701120; doi:10.3390/ijerph182412957)
Supplement: Supplementary file 1 [file ijerph-18-12957-s001.zip › ijerph-1442094-supplementary.pdf]

Supplementary materials

Table S1: Items pertaining to each socioeconomic deprivation domain

Socio-economic Deprivation Domain	Item Description
Nutritional deprivation (6 items)	Experience of skipping meals due to a lack of money (oneself or family)
	Experience of having no money to buy food even though there was nothing to eat
	Experience of unbalanced diet due to a lack of money to buy good food
	Experience of reducing or skipping meals (adults in the household) due to a lack of money
	Experience of eating less than one's appetite due to a lack of money
	Experience of suppressing hunger due to a lack of money to buy food
Housing deprivation (10 items)	A robust building with heat, fire, flame, and moisture resistance.
	Equipped with appropriate soundproofing, ventilation, lighting, and heating facilities
	Safe from natural disasters such as tsunamis, floods, landslides, and cliff collapses
	Unfit for living due to noise, vibrations, malodor, and air pollution
	Experience of being evicted due to rent being overdue for more than 2 months
	Experience of not being able to access heating in the winter due to a lack of money
	Rooftop/basement housing
	Sufficient rooms for household members: at least one room per two persons
	Sufficient space for the household:12 ㎡/1; 20 ㎡/2; 29 ㎡/3; 37 ㎡/4; 41 ㎡/5; 49 ㎡/6
	Meeting the minimum equipment standards for kitchen/toilet/bath facilities
Education deprivation (2 items)	Experience of not paying school fees (public education of children) for more than a month
	Experience of suspension of school due to difficulties in livelihood
Occupational/economic deprivation (4 items)	The total cost of living exceeding the minimum cost of living
	Temporary employment
	Unemployment
	Experience of working without safety facilities or in hazardous environments
Social security deprivation (5 items)	Experience of not having/paying for the National Pension Plan due to a lack of money
	Experience of suspension of insurance benefits due to defaulting on paying insurance premiums
	Non-subscription to Industrial Accident Compensation
	Non-subscription to Unemployment Insurance
	Non-application for severance pay

Social deprivation (4 items)	Experience of electricity, telephone, and water being shut-off due to not paying taxes<br>Experience of having a credit defaulter in the household<br>Dissatisfaction with family relationships<br>Dissatisfaction with social acquaintance network
Healthcare deprivation (3 items)	Experience of being unable to go to the hospital (oneself or family) due to a lack of money<br>History of suffering from at least one chronic disease<br>Dissatisfaction with health status

Table S2: Main variable correlations

	1	2	3	4	5	6	7	8	9	10	11	12	13	14	15	16
**1**	1															
**2**	-.153*	1														
**3**	.152*	-.067*	1													
**4**	-.222*	.217*	-.441*	1												
**5**	.132*	.079*	-.088*	.051*	1											
**6**	.287*	-.121*	.129*	-.207*	-.071*	1										
**7**	-.002	-.123*	.080*	.038*	-.035*	-.006	1									
**8**	.393*	-.066*	.139*	-.228*	.161*	.289*	-.001	1								
**9**	.059*	-.023	-.047*	.083*	.039*	.012	-.066*	-.047*	1							
**10**	.300*	-.005	.034*	-.062*	.071*	.141*	.047*	.283*	.024	1						
**11**	.301*	-.009	.048*	-.211*	.132*	.199*	.002	.295*	.034*	.248*	1					
**12**	-.002	.026	-.030	.059*	-.030	-.000	.030	-.010	.019	.084*	-.018	1				
**13**	.042*	-.039*	-.049*	-.060*	.060*	.104*	-.049*	.032*	.038*	.015	.080*	-.007	1			
**14**	.252*	-.150*	.227*	-.335*	.064*	.117*	.023	.267*	-.008	.117*	.174*	-.004	.068*	1		
**15**	.081*	-.009	-.002	.002	.037*	.078*	.002	.032*	.033*	.134*	.159*	.037*	.031*	.034*	1	

**16**	.359*	-.080*	.195*	-.244*	.040*	.155*	.017	.230*	.026	.157*	.198*	.017	.014	.228*	.035*	1

1. Depression, 2. Gender (male), 3. Age, 4. Education level, 5. Marital status (single) 6. Marital status (divorced/widowed/separated) 7. Residential area (capital area), 8. Poverty (low-income group), 9. Religion (Y/N) 10. Nutritional deprivation, 11. Housing deprivation, 12. Educational deprivation, 13. Occupational/economic deprivation, 14. Social security deprivation, 15. Social deprivation, 16. Healthcare deprivation

* $p<0.05$
